# *Tubuca alcocki*, a new pseudocryptic species of fiddler crab from the Indian Ocean, sister to the southeastern African *T. urvillei* (H. Milne Edwards, 1852) (Crustacea, Decapoda, Brachyura, Ocypodidae)

**DOI:** 10.3897/zookeys.747.23468

**Published:** 2018-03-29

**Authors:** Hsi-Te Shih, Benny K.K. Chan, Peter K.L. Ng

**Affiliations:** 1 Department of Life Science and Research Center for Global Change Biology, National Chung Hsing University, 250, Kuo Kuang Road, Taichung 402, Taiwan; 2 Biodiversity Research Center, Academia Sinica, Taipei 115, Taiwan; 3 Lee Kong Chian Natural History Museum, National University of Singapore, 6 Science Drive 2, Singapore 117546, Republic of Singapore

**Keywords:** mitochondrial cytochrome oxidase subunit I, molecular clock, morphology, new species, *Tubuca alcocki*, *Tubuca urvillei*

## Abstract

A new pseudocryptic species of fiddler crab, *Tubuca alcocki* **sp. n.**, is described from the northern Indian Ocean. The new species was previously identified with *T. urvillei* (H. Milne Edwards, 1852), but can be distinguished by the structures of the anterolateral angle of the carapace and male first gonopod. The molecular data of the mitochondrial cytochrome oxidase subunit I gene shows that both are sister taxa and the divergence time is estimated at 2.2 million years ago, around the beginning of the Pleistocene. While the new species is widely distributed in the northern part of Indian Ocean, occurring from the Red Sea to India and the Andaman Sea; *T. urvillei* sensu stricto has a more restricted range, and is known only from southeastern Africa.

## Introduction

In recent years, various genetic and morphological studies on fiddler crabs (Ocypodidae) from the Indian Ocean have shown that there are a number of species endemic to the region: *Austruca albimana* (Kossmann, 1877), *A. bengali* (Crane, 1975), *A. iranica* (Pretzmann, 1971), *A. occidentalis* (Naderloo, Schubart & Shih, 2016), *A. sindensis* (Alcock, 1900), *Cranuca inversa* (Hoffmann, 1874), *Paraleptuca chlorophthalmus* (H. Milne Edwards, 1837), *Gelasimus hesperiae* (Crane, 1975), and *Tubuca urvillei* (H. Milne Edwards, 1852) (Shih et al. 2009, 2010, 2012, 2013a,b, 2015, 2016; Naderloo et al. 2016). The genetics suggest that the cladogenesis of these taxa have their origins in the Indian Ocean.

Of these taxa, *Tubuca urvillei* is a large-sized species, which has been widely reported from throughout the Indian Ocean and is the only *Tubuca* Bott, 1973 species known in the western Indian Ocean (Crane 1975; Shih et al. 2016). Aspects of its biology has also been investigated in southeastern Africa (e.g., Macnae 1963; Hartnoll 1975; Litulo 2005; Peer et al. 2015), Thailand (e.g., Jaroensutasinee et al. 2003; Jaroensutasinee and Jaroensutasinee 2004), and Pakistan (Ghory and Siddiqui 2006).

In this study, specimens from the range of *Tubuca urvillei*, including the type specimens, were examined. There are two clades, with small but consistent morphological differences supported by DNA evidence from cytochrome oxidase subunit I (COI). The material from the northern and eastern parts of the Indian Ocean is herein described as a new pseudocryptic species, *T. alcocki*.

## Materials and methods

Specimens of *Tubuca urvillei* sensu lato collected from southeastern Africa, India and western Thailand examined (including the types) are deposited in the Muséum national d’Histoire naturelle, Paris, France (**MNHN**); Zoological Collections of the Department of Life Science, National Chung Hsing University, Taichung, Taiwan (**NCHUZOOL**); Senckenberg Museum, Frankfurt am Main, Germany (**SMF**); and Zoological Reference Collection of the Lee Kong Chian Natural History Museum (formerly Raffles Museum of Biodiversity Research), National University of Singapore, Singapore (**ZRC**). The abbreviation G1 is used for male first gonopod. Measurements, all in millimeters (mm), are of the maximum carapace width (**CW**), carapace length (**CL**) and pollex length (**PL**). The terminology used essentially follows Crane (1975) and Davie et al. (2015).

Sequences of COI were obtained following the method described by Shih et al. (2016), after verification with the complimentary strand. Sequences of the different haplotypes have been deposited in the DNA Data Bank of Japan (**DDBJ**) (accession numbers in Table 1). According to Shih et al. (2016), *T. urvillei* is sister to the clade composed of *T. dussumieri* (H. Milne Edwards, 1852), *T. paradussumieri* (Bott, 1973) and *T. capricornis* (Crane, 1975). As a result, the sequences of these three species, as published in Shih et al. (2016) (LC150436, LC053373 and LC150430), are used as outgroups in this paper.

**Table 1.** The haplotypes of COI gene of *Tubuca alcocki* sp. n. and *T. urvillei* from the Indian Ocean. Abbreviations of museums or universities see Material and methods.

Species	Locality	Catalogue no.	Sample size	Access. no. of COI
*Tubuca alcocki* sp. n.	India: Mumbai	NCHUZOOL 14899, 14925, 14901, 14902	4	LC150445
		NCHUZOOL 14903	2	LC150445
	Thailand: Ranong	ZRC (paratype)	2	LC369625
		NCHUZOOL 14896 (paratype)	1	LC369625
		ZRC 2017.1278 (holotype)	1	LC369625
	Thailand: Phuket	ZRC 1999.1131	1	LC369625
		ZRC 1999.1131	1	LC369626
*Tubuca urvillei*	Mayotte: Poroani	ZRC 1999.1107	1	LC053375
	Kenya: Shimo la Tewa	SMF 19985	1	LC053375
	Kenya: Mida Creek, Malindi	NCHUZOOL 14895	1	LC053375

The phylogenetic tree was reconstructed by the maximum likelihood (**ML**) analysis by using RAxML (vers. 7.2.6, Stamatakis 2006), with the model GTR + G (i.e. GTRGAMMA) was used with 100 runs, and found the best ML tree by comparing the likelihood scores. The robustness of the ML tree was evaluated by 1000 bootstrap pseudoreplicates under the model GTRGAMMA. Basepair (bp) difference, as well as the pairwise estimates of Kimura 2-parameter (**K2P**) distance (Kimura 1980) and the uncorrected p-distance for genetic diversities between haplotypes were also calculated by MEGA (vers. 7.0, Kumar et al. 2016).

## Systematic account

### Family Ocypodidae Rafinesque, 1815

#### Subfamily Gelasiminae Miers, 1886 (*sensu* Shih et al. 2016)

##### Genus *Tubuca* Bott, 1973

###### Tubuca urvillei

Taxon classificationAnimaliaDecapodaOcypodidae

(H. Milne Edwards, 1852)

**Figure 1.**
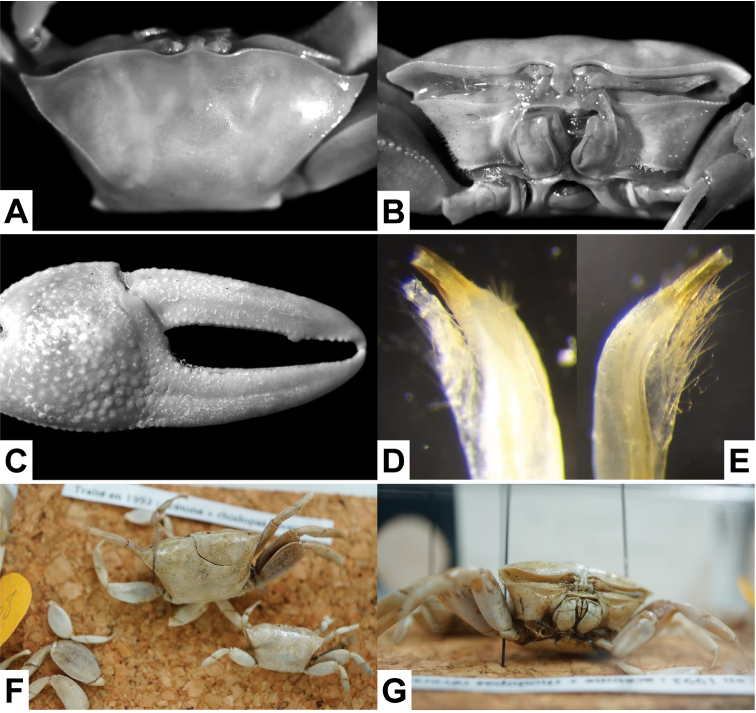
*Tubuca urvillei* (H. Milne Edwards, 1852). **A–E** lectotype (CW 18.5 mm, PL 17 mm, MNHN B.12073) **F–G** 2 dried female paralectotypes (MNHN B. 3208). **A** dorsal view **B** frontal view **C** major cheliped **D, E** distal part of right G1. **D** mesial view **E** lateral view.

**Figure 2.**
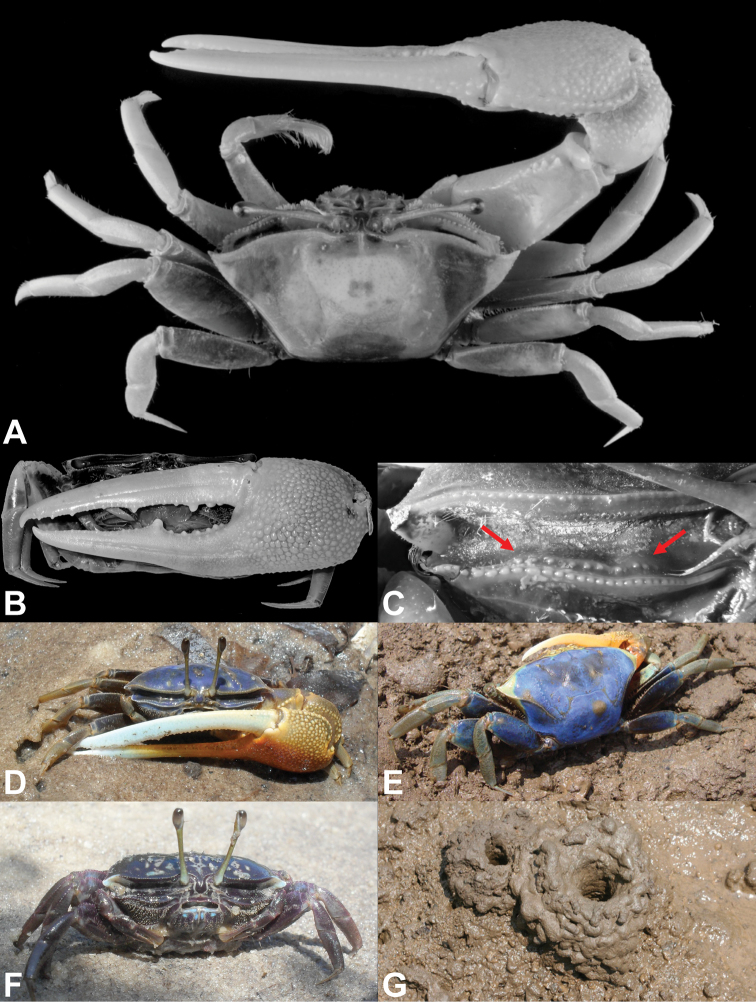
*Tubuca urvillei* (H. Milne Edwards, 1852) **A** male (CW 29.7 mm, ZRC 1999.1107; Mayotte) **B–C** male (CW 34.9, NCHUZOOL 14895; Kenya). **A** dorsal view **B** major cheliped **C** floor of right orbit of showing the tubercles (arrowed). **D–F** live colouration. **D** male from Kenya **E** male from Mayotte **F** female from Kenya **G** chimneys built by *T. urvillei* in Mayotte **D, F** courtesy of S. Cannicci **E, G** courtesy of J. Poupin.

Figures 1 , 2 , 4B , 5E–H , 7B, D, F, H

Gelasimus arcuatus – Krauss 1843: 39 [Natal Bay, South Africa] (not Ocypode (Gelasimus) arcuata De Haan, 1835).
Gelasimus urvillei H. Milne Edwards, 1852: 148, pl. 3(10) [type locality: “Vanikoro”]; Kingsley 1880: 145 [list]; De Man 1891: 21, 34 [Nossy Faly, Madagascar]; Ortmann 1894: 59 [Dar es Salaam, Tanzania].
Gelasimus dussumieri – A. Milne-Edwards 1868: 71 [list; Zanzibar]; Hilgendorf 1869: 84, pl. 4(1) [Zanzibar]; Hoffmann 1874: 17–18, pl. 3(19–22) [part; Nossy Faly, Madagascar]; De Man 1880: 68 [part; Madagascar]; Kingsley 1880: 145 [list; part]; Lenz and Richters 1881: 423 [Madagascar]; Pfeffer 1889: 30 [Zanzibar]; De Man 1891: 20, 26 [part; Nossy Faly, Madagascar]; Lenz 1910: 559 [Zanzibar; Pemba] (not Gelasimus dussumieri H. Milne Edwards, 1852).
Uca arcuata – Stebbing 1905: 40 [South Africa]; Stebbing 1910: 327 [list] (not Ocypode (Gelasimus) arcuata De Haan, 1835).
Uca arcuatus – Stebbing 1917: 15 [Natal, South Africa] (not Ocypode (Gelasimus) arcuata De Haan, 1835).
Uca dussumieri – Maccagno 1928: 17–19 [part; Giumbo, Somalia] (not Gelasimus dussumieri H. Milne Edwards, 1852).
Uca urvillei – Barnard 1950: 93–94, figs 18d–f, 19a–b; Fourmanoir 1954: 3 [Madagascar]; Macnae 1963: 23 [Inhaca I., Mozambique to Cape Province, South Africa]; Richmond 1997: 226, 2 unnumbered figs on p. 227 [eastern Africa]; Crosnier 1965: 110–112, figs 186, 191–193, 195–196; Kensley 1981: 49 [list]; Rosenberg 2001: 860, 868 [South Africa]; Serbino 2008: 62–72, fig. 1 [Mozambique].
Tubuca urvillei – Bott 1973: fig. 11; Shih et al. 2016: 159 [list; part].
Uca (Uca) urvillei – Hartnoll 1975: 308, 310, 322, 324, fig. 8 [Tanzania].
Uca (Uca) dussumieri – Hartnoll 1975: 308, 310 [list; Tanzania] (not Gelasimus dussumieri H. Milne Edwards, 1852).
Uca (Deltuca) [*coarctata*] urvillei – Crane 1975: 58–61, figs 7, 8D, 9D, 27G–H, 38U–X, 62E, 75, pl. 9A–B, E–H [part, southeastern Africa].
Uca (Deltuca) urvillei – Vannini and Valmori 1981: 212–213, figs 5F1, F2, 6F [Giumbo, Somalia].
Uca (Tubuca) urvillei – Bouchard et al. 2013: 46, fig. 40 (Mayotte); Peer et al. 2014: 60, fig. 15; 2015: 190, 198, fig. 4c, d (upper figure) [South Africa].

####### Material examined.

Lectotype ♂ (CW 18.5 mm, CL 11.0 mm, PL 17.0 mm) (MNHN B.12073), “Vanikoro”, coll. J. R. C. Quoy and J. P. Gaimard (Fig. 1A–E). Paralectotypes: 2 ♀♀ (MNHN B. 3208), same data as lectotype (Fig. 1F–G).

####### Other material.

1 ♂ (CW 28.5 mm), 1 ♀ (CW 22.9 mm) (SMF 19985), Shimo la Tewa, ca. 20 km N Mombasa, ca. 2 km von Küste entfernt, Schlickmangrove, Kenya, coll. H. Langer, 11 Aug. 1990; 1 ♂ (CW 29.7 mm) (ZRC 1999.1107), Poroani, mangrove to the south, Mayotte, 23 July 1998; 2 ♂♂ (CW 27.9–34.9 mm), NCHUZOOL 14895, Mida Creek, Malindi, Kenya.

####### Diagnosis.

**Male**. Carapace (Figs 1A, 2A, 4B, 7B, D, F) with anterolateral angle (= external orbital angle) broadly triangular, directed laterally; anterolateral margin short to moderately long; dorsolateral margin long, definite, strongly converging; 1 posterolateral stria. Floor of orbit with row of fewer than 17 tubercles, sometime with blunt ridge (Figs 1B, 2C, D). Major cheliped (Figs 1C, 2B) with dactylus usually longer than palm, outer surface of dactylus and pollex each with 1 long groove proximally extending beyond midlength. Fingers of minor cheliped without conspicuous tooth on either finger. G1 (Fig. 5E–H) with distal tube relatively stout, distinctly curved, gently tapering towards tip, distal part distinctly narrower than proximal part; thumb of moderate length, extending beyond base of distal tube. **Female**. Carapace with anterolateral angle acutely triangular; anterolateral margin short or absent, joining dorsolateral margin as almost straight line (Fig. 7H). Floor of orbit with row of 14–16 tubercles (Fig. 2F). Fingers of cheliped (Fig. 2F) each with conspicuous tooth on occlusal margin. (See also Remarks under *T. alcocki* sp. n. for comparisons of morphology and colouration.)

####### Distribution.

Southeastern Africa from Giumbo (= Jumboo), southern Somalia, to Cape Province, South Africa (mouth of Umtata R.); Madagascar (Crane 1975).

####### Remarks.

In his revision of the genera and subgenera of the fiddler crabs of the world, Bott (1973) established *Tubuca* and designated *Gelasimus urvillei* H. Milne Edwards, 1852 as the type species from the lectotype (Bott 1973: fig. 11). The type specimens of *Tubuca urvillei* were supposedly collected from “Vanikoro” (an island between Solomon and Vanuatu) in the western Pacific. Crane (1975) queried this type locality noting that the species as she understood it did not occur outside the Indian Ocean. As such, Crane (1975) considered the data on the label to be wrong. Of the three extant type specimens of *Gelasimus urvillei* H. Milne Edwards, 1852, Crane (1975) selected the male as the lectotype, the other two females becoming paralectotypes (Fig. 1F–G). Crane (1975) considered the male to be an immature specimen (CW 18.5 mm) but its G1 is actually already developed (present study). According to Litulo (2005), the smallest ovigerous female from Mozambique is only CW 10.0 mm. This suggests that the lectotype male, while small is probably already mature. In any case, the G1 of the lectotype of *T. urvillei* (Crane 1975: fig. 9D) agrees well with the species as is now understood from southeastern Africa (cf. Fig. 5E–H). They also agree in all other morphological characters.

A note on *Gelasimus dussumieri* H. Milne Edwards, 1852 (at present *Tubuca dussumieri*) is necessary. The type material of *Tubuca dussumieri* include specimens from Samarang (Java, Indonesia) and Malabar (Mumbai, India) (H. Milne Edwards, 1852), and as no holotype was originally selected, Crane (1975) designated a male from Samarang as the lectotype of *T. dussumieri*. The paralectotype male from Malabar, however, she reidentified as *T. urvillei* instead. She also found that *T. dussumieri* and *T. paradussumieri* were sympatric in the western Pacific and eastern part of Indian Ocean. She reidentified all the records (including “*T. acuta*”) from western Indian Ocean as *T. urvillei*, with one exception – the record of *G. dussumieri* by Hoffmann (1874: pl. 3(22)) and De Man (1891) from Nossy Faly, northern Madagascar, which was referred to *T. paradussumieri* instead. As no other record of *T. paradussumieri* from eastern Africa has been reported since 1874 (Crosnier 1965), Crane (1975) regarded this specimen’s provenance as questionable. Another record of “*T. dussumieri*” from Bombay, western India (Krishnan 1992) will also need to be confirmed in the future as well. In summary, Crane (1975) emphasized the westernmost distribution of the genus *Tubuca* (= *Deltuca* Crane, 1975) should be *T. urvillei* from southeastern Africa (Tanzania, Madagascar and South Africa), with the species also present in Pakistan and western India. Later, the species was reported from the Red Sea by Hogarth (1986) and Price et al. (1987).

With regard to the records of *T. urvillei* and *T. acuta* in Alcock (1900), Crane (1975: 61) considered only those from Pakistan and western India as belonging to true *T. urvillei* (shown as “(part)” behind these records). That is, she did not think or was uncertain if the records from the Bay of Bengal and the Andaman Sea (e.g. Madras; Sunderbunds; Mergui; Andamans and Nicobars) by Alcock (1900) were also *T. urvillei*. Lundoer (1974) added a new record of “*U. angustifrons* (De Man, 1892)” from Phuket, Thailand, but this was later reidentified as *T. urvillei* by Frith et al. (1976) and Frith and Frith (1977a) (see also Frith and Frith 1978; Frith and Brunenmeister 1980, 1983; Jaroensutasinee et al. 2003; Jaroensutasinee and Jaroensutasinee 2004).

###### Tubuca alcocki sp. n.

Taxon classificationAnimaliaDecapodaOcypodidae

http://zoobank.org/0912FA92-20A2-424F-82C0-A337A20A4494

**Figure 3.**
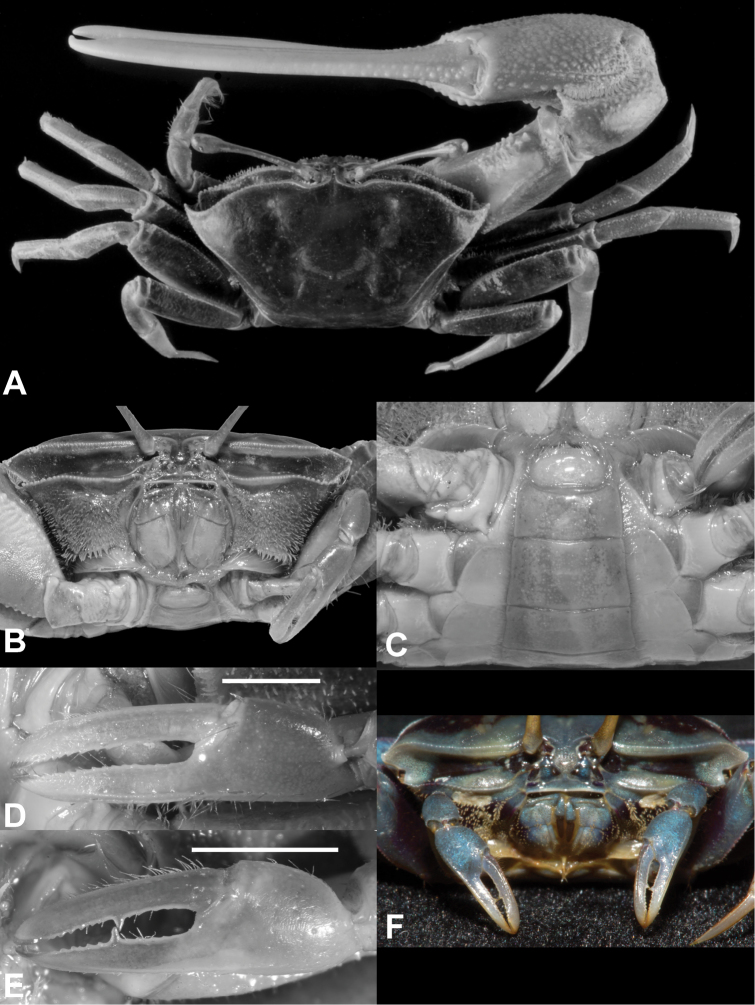
*Tubuca alcocki* sp. n. **A–D** holotype (CW 30.1 mm, ZRC 2017.1278) **E, F** ovigerous female (CW 19.8 mm, NCHUZOOL 14897; Thailand). **A** dorsal view **B** frontal view **C** pleon and telson **D, E** left minor cheliped **F** frontal view, with living colouration. Scale bars: 5.0 mm.

**Figure 4.**
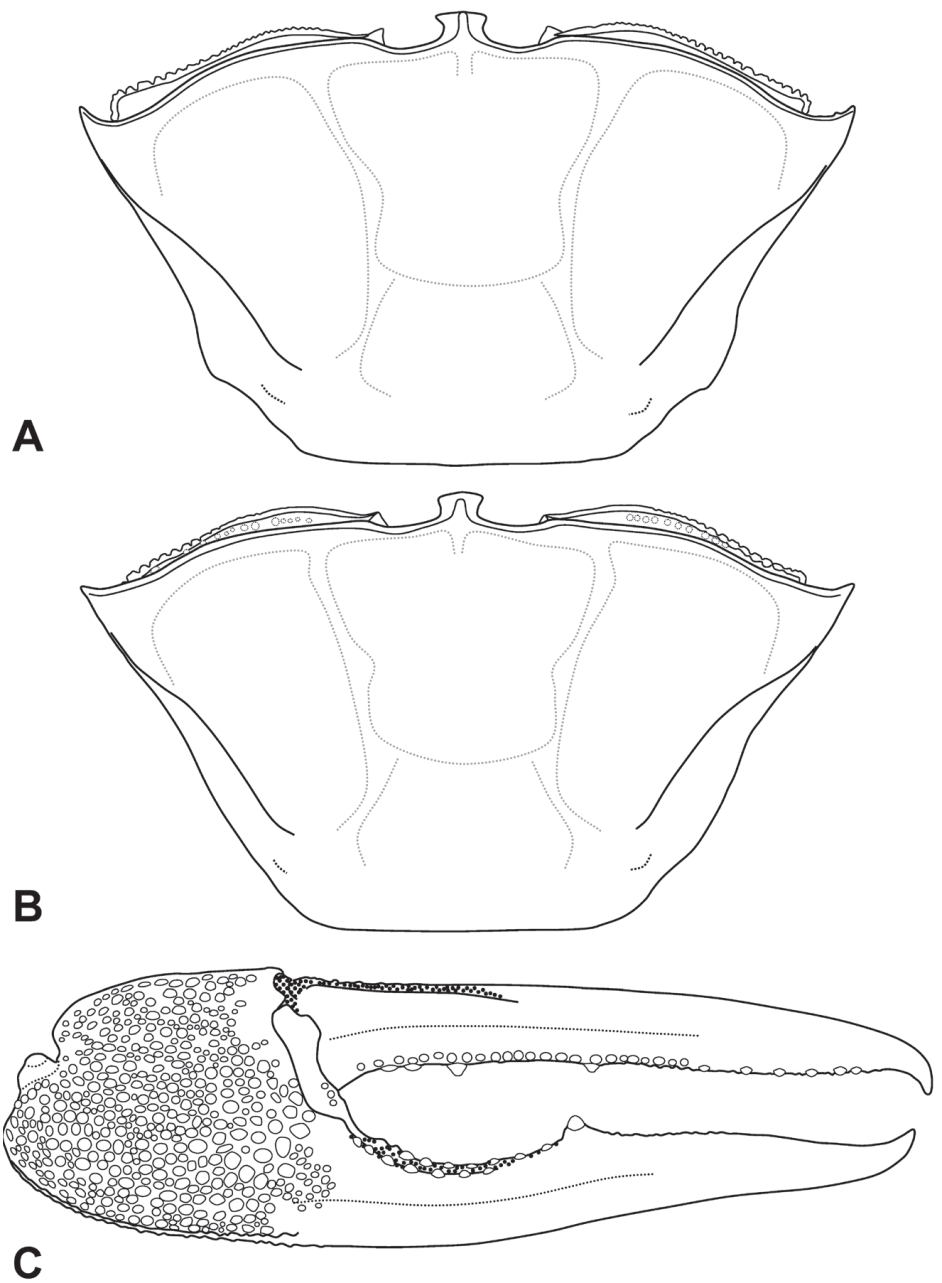
*Tubuca alcocki* sp. n. **A, C** holotype (CW 30.1 mm, PL 58.2 mm, ZRC 2017.1278; Thailand); *T. urvillei* (H. Milne Edwards, 1852) **B** male (CW 29.7 mm, ZRC 1999.1107; Mayotte). **A, B** dorsal view **C** major cheliped.

**Figure 5.**
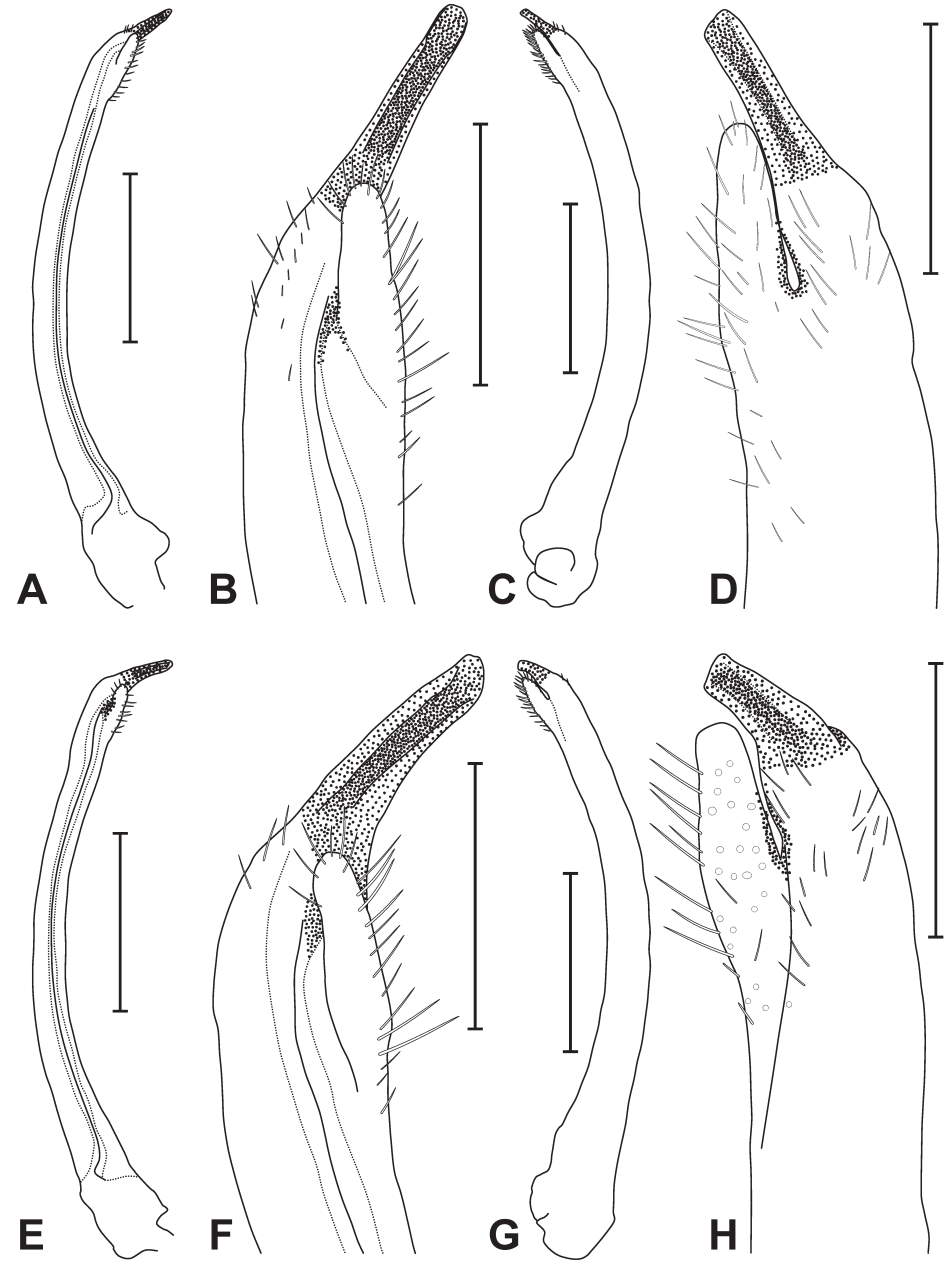
Right G1. *Tubuca alcocki* sp. n. **A–D** holotype (CW 30.1 mm, ZRC 2017.1278; Thailand) **E–H** *T. urvillei* (H. Milne Edwards, 1852), male (CW 29.7 mm, ZRC 1999.1107; Mayotte). **A, E** mesial view **B, F** mesial view of distal part **C, G** lateral view **D, H** lateral view of distal part. Scale bars: **A, C, E, G** 5.0 mm **B, D, F, H** 1.0 mm.

**Figure 6.**
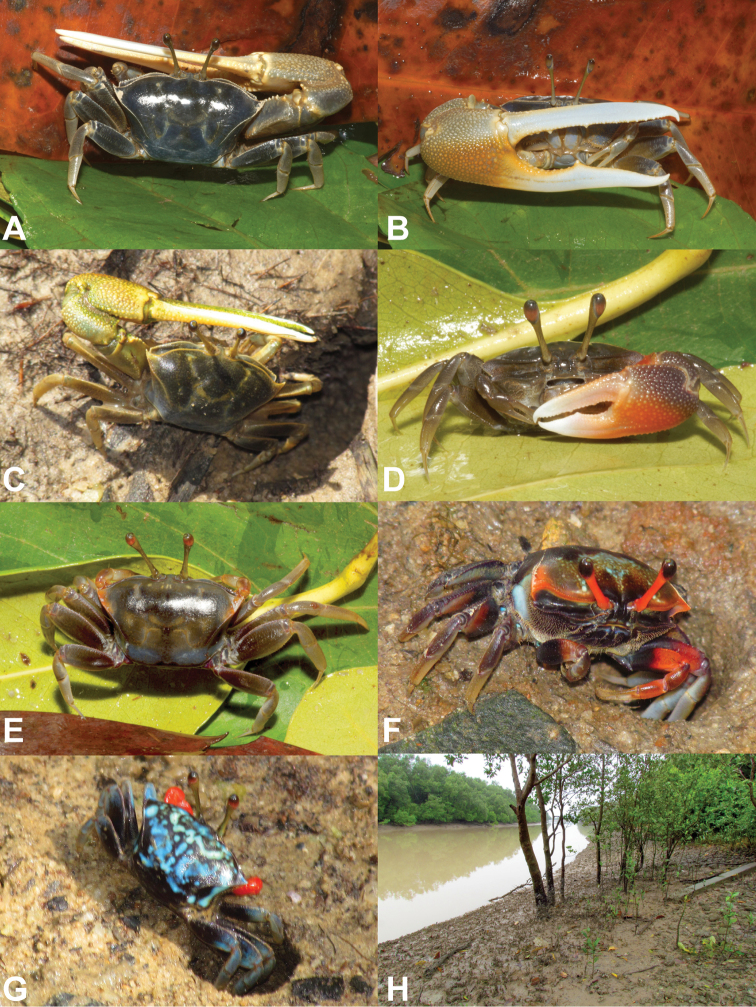
*Tubuca alcocki* sp. n. **A–G** variation of the live colouration. **A, B** holotype (CW 30.1 mm, ZRC 2017.1278; Thailand) **C** adult male (not collected; Phuket, Thailand) **D** young male (CW 13.0 mm, NCHUZOOL 14897; Thailand) **E** ovigerous female (CW 19.8 mm, NCHUZOOL 14897, Thailand) **F, G** females in the field (not captured; Phuket, Thailand) **H** habitat in Ranong, Thailand.

**Figure 7.**
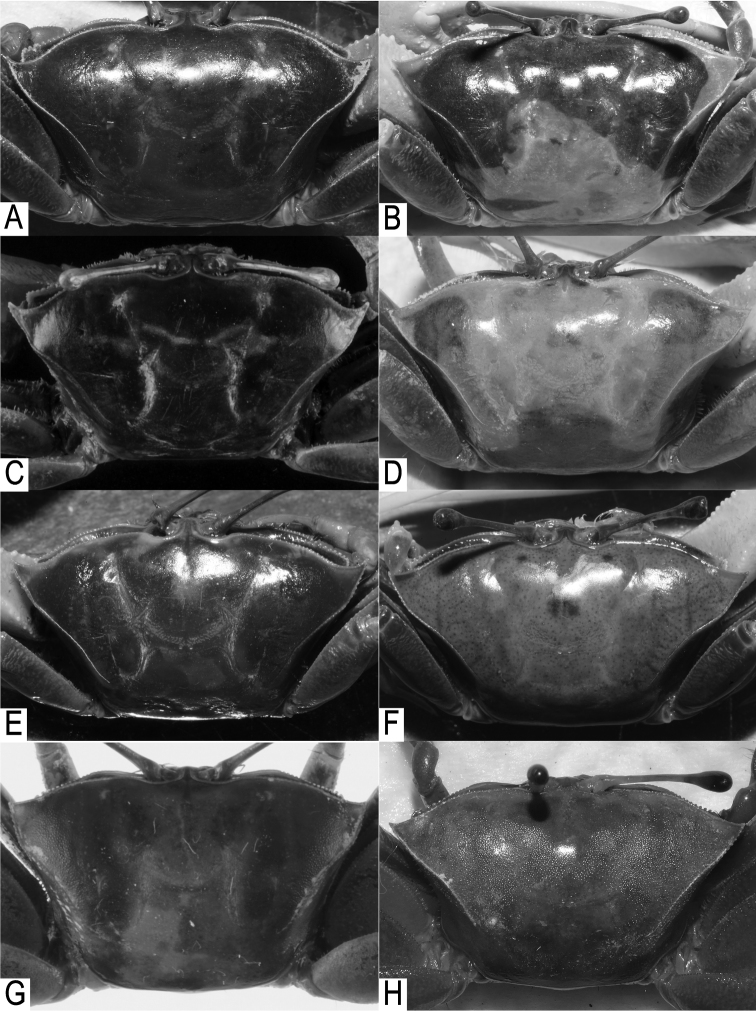
Carapace morphology. **A, C, E, G** *Tubuca alcocki* sp. n., **B, D, F, H** *T. urvillei* (H. Milne Edwards, 1852). **A** holotype male (CW 30.1 mm, ZRC 2017.1278; Thailand) **B** male (CW 34.9 mm, NCHUZOOL 14895; Kenya) **C** male (CW 29.5 mm, NCHUZOOL 14896; Ranong, Thailand) **D** male (CW 29.7 mm, ZRC 1999.1107; Mayotte) **E** male (CW 27.8 mm, NCHUZOOL 14905; Thailand) **F** male (CW 27.9 mm, NCHUZOOL 14895; Kenya) **G** female (CW 22.6 mm, NCHUZOOL 14900; India) **H** female (CW 22.9 mm, SMF 19985; Kenya).

Figures 3 , 4A, C , 5A–D , 6 , 7A, C, E, G

**Figure 8.**
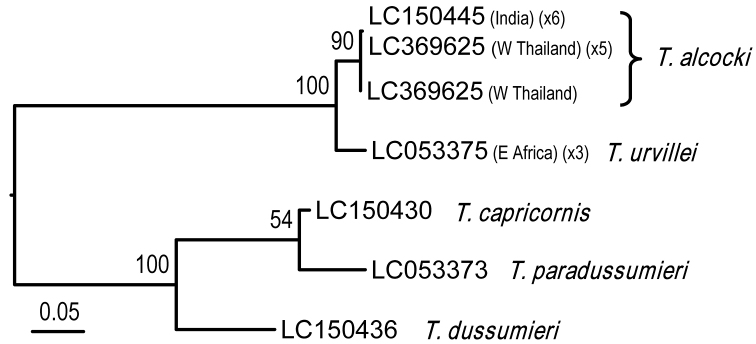
A maximum likelihood (ML) tree for *Tubuca urvillei* (H. Milne Edwards, 1852) and *T. alcocki* sp. n. from the Indian Ocean, and outgroups, based on COI gene. Bootstrap proportions are shown at the nodes. For accession numbers, see Table 1 and Materials and methods.

Gelasimus Dussumieri H. Milne Edwards, 1852: 148, pl. 4(12) [part; Malabar, India]; Kingsley 1880: 145 [part; list]; Chandy 1973: 402 [Gulf of Kutch, W India] (not Gelasimus dussumieri H. Milne Edwards, 1852 sensu stricto).
Gelasimus acutus – Alcock 1900: 360–361 [Sunderbunds, Mergui; Andamans; Karachi] (not Gelasimus acutus Stimpson, 1858).
Gelasimus Urvillei – Alcock 1900: 362–363 [Nicobars; Madras; Karachi] (not Gelasimus urvillei H. Milne Edwards, 1852).
Uca angustifrons – Lundoer 1974: 8 [Phuket, SW Thailand]; Ng and Davie 2002: 378 [list; Phuket, SW Thailand] (not Gelasimus signatus var. angustifrons De Man, 1892 = Tubuca bellator (White, 1847)).
Uca (Deltuca) [*coarctata*] urvillei – Crane 1975: 35, 58–61, figs 8B, 9E, pl. 9C, D [part, Pakistan to southern India]; Frith and Frith 1977a: 100–101 [Phuket, SW Thailand] (not Gelasimus urvillei H. Milne Edwards, 1852).
Uca urvillei – Frith et al. 1976: 14, 19, 23–24, 28 [Phuket, SW Thailand]; Tirmizi and Ghani 1996: 103–105, fig. 39 [Pakistan]; Jaroensutasinee et al. 2003: 1–3 [W Thailand]; Jaroensutasinee and Jaroensutasinee 2004: 534, 538, 540–548 [W Thailand]; Naiyanetr 2007: 133 [list; Thailand]; Saher 2008: 21–22, fig. 2.2, pl. 2.1 [Pakistan]; Dev Roy and Nandi 2012: 218 [Nicobar, India]; Hossain 2015: 203, 1 unnumbered fig. [Bangladesh]; Odhano et al. 2015: 170–171, figs 1–2 [Pakistan] (not Gelasimus urvillei H. Milne Edwards, 1852).
Uca (Deltuca) urvillei – Hogarth 1986: 222–223 [Red Sea]; Price et al. 1987: 456, 464 [Red Sea]; Krishnan 1992: 471–472 [Bombay, India] (not Gelasimus urvillei H. Milne Edwards, 1852).
Uca (Deltuca) dussumieri – Krishnan 1992: 471–472 [Bombay, India] (not Gelasimus dussumieri H. Milne Edwards, 1852)
Uca (Tubuca) urvillei – Beinlich and von Hagen 2006: 10, 14, 25, fig. 7f, k [Thailand; India] (not Gelasimus urvillei H. Milne Edwards, 1852).
Uca (Tubuca) acuta – Trivedi et al. 2015: 27 [Gujarat, India] (not Gelasimus acutus Stimpson, 1858).
Tubuca urvillei – Shih et al. 2016: 159, 174 [part], fig. 12A.

####### Material examined.

Holotype: ♂ (CW 30.1 mm, CL 17.9 mm; PL 58.2 mm) (ZRC 2017.1278), Ranong mangroves, Thailand, coll. H.-T. Shih et al., 27 May 2012. Paratypes: 2 ♂♂ (CW 22.4–29.9 mm), 1 ♀ (CW 25.1 mm) (NCHUZOOL 13661), 1 ♂ (CW 29.5 mm) (NCHUZOOL 14896), 13 ♂♂ (CW 14.7–31.2 mm), 4 ♀♀ (CW 19.9–24.1 mm), 1 ovig. ♀ (CW 25.7 mm) (NCHUZOOL 14905), same data as holotype; 1 ♂ (CW 24.6 mm), 1 ovig. ♀ (CW 14.8 mm) (ZRC 2017.1279), Kamphuan mangroves, Ranong, Thailand, 9 Sep. 2000; 1 ♂ (CW 24.0 mm) (ZRC 2001.2347), Ranong, Thailand, coll. P. Clark, 7 Nov. 2001.

####### Other material.

Thailand: 2 ♂♂ (CW 17.8–26.1 mm) (ZRC 1988.616–617), Phuket, coll. D. H. Murphy, 12 Nov. 1987; 2 ♂♂ (CW 20.3–22.4 mm) (ZRC 1999.1131), mangrove area south east of Phuket Town ca. 8 km, W. B. Jeffries and H. K. Voris, 14 June 1990; 4 ♂♂ (CW 13.0–16.9 mm), 1 ♀ (CW 18.8 mm), 1 ovig. ♀ (CW 19.8 mm) (NCHUZOOL 14897), 6 ♂♂ (CW 9.0–12.4 mm), 3 ♀♀ (CW 11.4–14.0 mm), 2 ovig. ♀♀ (CW 13.9–14.8 mm) (NCHUZOOL 14906), Chalong Bay, Phuket, coll. H.-T. Shih et al., 28 May 2012; 4 ♂♂ (CW 4.4–16.9 mm), 1 ♀ (CW 12.8 mm), 1 ovig. ♀ (CW 14.7 mm) (NCHUZOOL 14898), Laem Tukkae, Phuket, coll. H.-T. Shih et al., 29 May 2012; 1 ♂ (CW 17.2 mm), 2 ♀♀ (CW 14.0–15.6 mm) (NCHUZOOL 14907), Tha Thiap Ruea Bang Rong, Phuket, coll. H.-T. Shih et al., 30 May 2012. India: 1 ♂ (CW 17.7 mm) (NCHUZOOL 14925), 1 ♂ (CW 19.0 mm) (NCHUZOOL 14899), 1 ♂ (CW 12.6 mm) (NCHUZOOL 14901), 1 ♀ (CW 17.5 mm) (NCHUZOOL 14902), 13 ♂♂ (CW 9.9–18.2 mm), 3 ♀♀ (CW 11.4–17.9 mm), 1 ovig. ♀ (CW 19.9 mm) (NCHUZOOL 14903), Mumbai, coll. H.-N. Chen et al., 17 Mar. 2010; 1 ♀ (CW 22.6 mm) (NCHUZOOL 14900), Diu mangroves, coll. K. Wong, 20 Mar. 2010.

####### Diagnosis.

**Male.** Carapace (Figs 3A, 4A, 6A, C, G, 7A, C, E) trapezoidal, smooth; front narrow, with distinct, narrow median groove; anterolateral angle acutely triangular, directed obliquely anteriorly; anterolateral margin short to moderately long; dorsolateral margin long, definite, strongly converging; one posterolateral stria. Floor of orbit with row of 5–11 tubercles, sometimes with blunt ridge. Major cheliped (Figs 4C, 6B, D) with dactylus usually longer than palm, outer surface of dactylus and pollex each with 1 long groove proximally extending beyond midlength. Fingers of minor cheliped (Figs 3B, D, 6B, D) without conspicuous tooth on either finger. G1 (Fig. 5A–D) with distal tube slender, slightly curved to almost straight, distal and proximal parts subequal in width; thumb of moderate length, extending beyond base of distal tube.

**Female.** Anterolateral angle more broadly triangular; anterolateral margin moderately long, joining dorsolateral margin as convex structure (Fig. 7G). Floor of orbit with row of 17–19 tubercles (Figs 3F, 6F). Fingers of cheliped (Fig. 3E, F) each with conspicuous tooth on occlusal margin.

####### Colouration in life.

Adults with carapace and legs brown or dark brown, posterior part gray, especially in females (Fig. 6A, C, E). Some females with anterolateral angles orange (Fig. 6E, F) or with dark blotches on blue carapace (Fig. 6G). Major cheliped with fingers white; lower palm deep yellow in large individuals, orange in young individuals; upper palm brown (Fig. 6B–D). Females sometimes with minor chelipeds orange, sometimes with tint of blue (Figs 3F, 6F, G).

####### Ecological notes.

In western Thailand, this species inhabits muddy banks of mangroves (Fig. 6H) and is sympatric with several species of fiddler crabs, including *Austruca annulipes* (H. Milne Edwards, 1837), *A. bengali*, *Tubuca forcipata* (Adams & White, 1849) and *T. paradussumieri* (cf. Frith and Frith 1977a, 1978; this study). In Pakistan, this species is sympatric with *Austruca iranica* (cf. Saher et al. 2014).

####### Etymology.

This species is named after Alfred William Alcock, who first recorded this species from India and Pakistan as “*Uca urvillei*” (cf. Alcock 1900).

####### Distribution.

Western Thailand, India, Pakistan, and the Red Sea (see Remarks).

####### Remarks.

Although the number of tubercles on the floor of orbit and thumb morphology of G1 are sometimes useful for distinguishing species of fiddler crabs, they are too variable in *Tubuca alcocki* sp. n. and *T. urvillei* (Crane 1975: 58–59; this study) to be used. The two species are similar, but can be morphologically distinguished by the characters of the anterolateral angle of the carapace and G1. The anterolateral angle in male *T. alcocki* is acutely triangular and directed obliquely anteriorly (Fig. 7A, C, E) (vs. relatively broadly triangular in shape and directed more laterally in position in *T. urvillei*; Fig. 7B, D, F). In female *T. alcocki*, the anterolateral angle is broadly triangular in shape and the anterolateral margin is relatively longer and curves gently to join the dorsolateral margin (Fig. 7G) (vs. anterolateral angle acutely triangular in shape with the anterolateral margin short and merging with the dorsolateral margin in an almost straight line in *T. urvillei*; Fig. 7H). The G1 structure is also different. The distal tube of the G1 of *T. alcocki* is proportionately more slender, being slightly curved to almost straight, with the widths of the distal and proximal parts subequal (Fig. 5A–D) (vs. distal tube relatively stouter, more distinctly curved and gently tapering towards the tip, with the distal part distinctly narrower than the proximal part in *T. urvillei*; Fig. 5E–H).

Crane (1975) figured specimens of what she referred to as *T. urvillei* from southeastern Africa and western India, and they agree with the characters of *T. urvillei* and *T. alcocki*, respectively. The anterolateral angles of the male lectotype of *T. urvillei* (Fig. 1A; Crane 1975: pl. 9E) and the male specimen from Tanzania (Crane 1975: fig. 7A) are both broadly triangular. In addition, the G1 distal tubes of the lectotype of *T. urvillei* as well as those from Somalia and Madagascar figured by Vannini and Valmori (1981: fig. 6F) and Crosnier (1965: figs 195–196) are all relatively stout, curved and tapering towards the tip. As such the material from Tanzania, Somalia and Madagascar should all be referred to *T. urvillei* sensu stricto.

The specimen from Malabar, western India, and one of the paralectotypes of *Gelasimus dussumieri* (see discussion earlier), have the G1 distal tube relatively more slender, almost straight, with the distal and proximal parts subequal in width (Crane 1975: fig. 9E) and are thus is clearly referable to *T. alcocki*. The G1 structures of specimens from Pakistan (Saher 2008: fig. 2.2; Tirmizi and Ghani 1996: fig. 39) also match that of *T. alcocki*. Interestingly, Hogarth (1986) reported “Uca (Deltuca) urvillei” from the Red Sea, which was a new record of this species for this region, but without any figure or description. The first author has examined specimens from the Red Sea and they are clearly *T. alcocki* as well (H-T Shih and BA Kumar, in preparation). The distribution of *T. alcocki* thus stretches from the northern part of the Indian Ocean (Red Sea) to the Arabian Sea and Andaman Sea.

There are also colour differences between *T. urvillei* and *T. alcocki*. While the colouration of females, young males, and juveniles are variable in *Tubuca* species, the colouration of the adult male carapace is generally more useful (Crane 1975; von Hagen and Jones 1989; Beinlich and von Hagen 2006). Adult male *T. urvillei* sensu stricto have various degrees of blue on the carapace and ambulatory legs (Fig. 2D, E), with the palm of the major cheliped ochraceous to apricot brown (Fig. 2D); while young and females sometimes have pale and dark blotches on a blue background (Fig. 2F). In adult male *T. alcocki*, the dorsal surface of the carapace is always dark brown (Fig. 6A, C) whereas in *T. urvillei*, it is always blue (Fig. 2D, E).

## DNA analyses and discussion

The molecular analyses include 12 specimens of *Tubuca alcocki* sp. n. from western Thailand and western India; and three specimens of *T. urvillei* from southeastern Africa (Table 1). The phylogenetic tree (Fig. 8) based on COI shows that specimens from southeastern Africa form a distinct clade, sister to another clade with material from western India and western Thailand. The genetics therefore supports the recognition of two species. Only one haplotype is found from *T. urvillei* from southeastern Africa, with two haplotypes from *T. alcocki*.

The genetic distance between these two sister species is 3.78 % (K2P distance) or 3.65 % (p-distance), and the total bp difference is 24 bp. The value is higher than some species within the Ocypodoidea, e.g., the minimum genetic distance of K2P between two species is 2.79 % between *Paraleptuca crassipes* (White, 1847) and *P. splendida* (Stimpson, 1858); 3.62 % between *Gelasimus hesperiae* (Crane, 1975) and “Clade U”; and 3.62 % between *Mictyris brevidactylus* Stimpson, 1858 and *M. guinotae* Davie, Shih & Chan, 2010, but still smaller than 6.25 % between *Ocypode stimpsoni* Ortmann, 1897 and *O. mortoni* George, 1982; and 4.43 % between *Scopimera globosa* (De Haan, 1835) and *S. ryukyuensis* Wong, Chan & Shih, 2010 (see Davie et al. 2010; Shih et al. 2010, 2012; Wong et al. 2010, 2012; Chu et al. 2015).

*Tubuca alcocki* and *T. urvillei* are quite similar in general morphology, but can be still distinguished by characters of the carapace and G1 (see Remarks under *T. alcocki*), which is supported by molecular evidence (Fig. 8). As a result, *T. alcocki* can be considered a pseudocryptic species (i.e., minor morphological difference, only after other methods have unveiled their existence), which is not uncommon in marine organisms (Knowlton 1993, 2000), including brachyuran crabs (Ragionieri et al. 2009, 2012; Shih et al. 2013a; Ng and Shih 2014; Lai et al. 2017).

According to Shih et al. (2016) and this study, the clade of *T. urvillei* and *T. alcocki* is sister to the clade composed of *T. dussumieri*, *T. paradussumieri* and *T. capricornis*. From the distributional patterns of these two main clades, while *T. urvillei* and *T. alcocki* are found only in the Indian Ocean, another main clade, composed of *T. dussumieri*, *T. paradussumieri* and *T. capricornis* (Shih et al. 2016), is primarily western Pacific, although *T. alcocki* and *T. paradussumieri* are sympatric in the eastern Indian Ocean (Frith and Frith 1977a, 1978; present study). Because *T. dussumieri* has been recorded from Surin Islands, Phang Nga Province, Thailand (Frith and Frith 1977a, b, 1978), *T. alcocki* is probably also sympatric with it.

Hogarth (1986) has emphasized that the Red Sea population of “*T. urvillei*” is discontinuous with other populations and represents a significant extension of the known range. His Red Sea material is now recognized as *T. alcocki* (unpublished data; see Remarks under *T. alcocki*), and the northernmost distribution of *T. urvillei* sensu stricto is in southern Somalia (Crane 1975; Hogarth 1986). *Tubuca alcocki* thus has a wider range, which includes most of the northern Indian Ocean, from western Thailand (facing the Andaman Sea), through the Bay of Bengal and India, to the Red Sea. *Tubuca urvillei*, on the other hand, is known with certainty only from southeastern Africa. This distributional pattern is probably caused by the major oceanographic circulation systems at around 10°S, which limit the dispersal of larvae to the southeastern African coastline (Tsang et al. 2012).

Based on the pairwise divergence rates of 1.66 % per million years for COI of marine coastal crabs (Schubart et al. 1998), *T. urvillei* and *T. alcocki* diverged 2.2±0.4 million years ago (mya) (with uncorrected p-distance divergences of 3.65 % ± 0.71 %) around the beginning of the Pleistocene. The divergence between them is probably caused by the change of larval dispersal routes through ocean currents (e.g., the Equatorial Counter Current for a biogeographic barrier of barnacles; Tsang et al. 2012), which was likely influenced by the extreme climate during the glaciation periods in the Pleistocene (Shih et al. 2013a).

## Supplementary Material

XML Treatment for Tubuca urvillei

XML Treatment for Tubuca alcocki
